# The Pathology of Emotions

**Published:** 1900-12

**Authors:** 


**The Pathology of Emotions.** By CH. FÉRÉ. Rendered into English by ROBERT PARK, M.D. Pp. vii., 525, xiv. London: The University Press, Ltd. 1899.

The author premises that the emotions as representative states of consciousness have physiological conditions similar to those aroused by the original excitations, and that physical agents which modify the one can, in like manner, modify the other. He then sets forth that this similitude of somatic states helps to establish the physical nature, normal or abnormal, of the mind, and to indicate that the study of mental medicine, as a specialisation of general medicine only, should be followed through physical procedures.

The physiology of attention is thoroughly examined and the discussion upon its relations to nutritional anomalies, functional paralysis, hysteria and mental disease occupies an extensive portion of the book. Plenty of space, too, is devoted to the physical changes accompanying morbid emotional states, to the reaction of mental and physical processes the one on the other, and to alterations in blood constitution and phagocytic activity. The chapters devoted to morbid psychology form a very important section of the book; and many other interesting matters are thoroughly discussed. The author speaks in terms of strong condemnation regarding the license given to the degenerate class by too free employment of the plea of irresponsibility; impunity for crimes and excess of assistance to all categories of degenerates concur, in his opinion, to traverse natural selection. He holds that too much confidence has been placed in the theory of the physical attributes of the degenerate class; the presence of deformities or maladies should not be allowed to bias the impartial administration of the law. The position frequently assumed by the medical expert in cases involving responsibility for crime is severely criticised. M. Féré would advocate increased rather than lessened stringency in dealing with the subjects of defective control, urging that discipline must correct the tendency to morbid impulses; this we believe to be sound reasoning, but we cannot agree with Féré in such a statement as the following:—"The most violent impulsions can be restrained so long as the subject has consciousness of his criminality of act and the consequences it may entail upon him" (p. 504).

Without having advanced any notable original matter, the author has treated his subject fully, adequately, and in an interesting manner, and the book should have been a distinct acquisition to the library of the psychological student. But before we can unhesitatingly recommend the purchase of the work, some strictures on the mode of its production in English form are imperative. The translation of a foreign work should only be entrusted to a proficient in the particular tongue, and if possible to one who has an especial knowledge of the subject treated. Dr. Park would seem to vouch for his own efficiency in the latter respect, inasmuch as he claims in the preface to have already arrived at Monsieur Féré's conclusions by other methods; from this the reader might hope to find an intelligent rendering of fairly straightforward French writing. But however erudite in psychology the translator may be, his labour is in vain if his knowledge of the language from which he translates is but scanty and his power to express his thoughts in his own language defective. For an example of Dr. Park's English style we indicate his own preface. For specimens of his faulty translation we refer the reader to the entire work, which is gravely prejudiced by blunders which a schoolboy would blush to commit. Such an unhappy rendering of an excellent book is the less excusable seeing that Dr. Park informs us that he has devoted his leisure to the task of translation.

